# Understanding the Orthopedic Conditions for Which Patients Are Seeking Medical Cannabis Certification

**DOI:** 10.7759/cureus.52829

**Published:** 2024-01-23

**Authors:** Juliet Chung, Yusuf Mahmoud, Sina Ramtin, Gianna Uhler, Asif M Ilyas, Ari Greis

**Affiliations:** 1 Orthopaedic Surgery, Penn State College of Medicine, Hershey, USA; 2 Orthopaedic Surgery, Rothman Orthopaedic Institute Foundation for Opioid Research & Education, Rothman Opioid Foundation, Philadelphia, USA; 3 Physical Medicine and Rehabilitation, Rothman Orthopaedic Institute at Thomas Jefferson University, Philadelphia, USA; 4 Orthopaedics, Rothman Orthopaedic Institute at Thomas Jefferson University, Rothman Opioid Foundation, Philadelphia, USA; 5 Department of Medical Cannabis, Rothman Orthopaedic Institute at Thomas Jefferson University, Philadelphia, USA

**Keywords:** orthopaedic, medical cannabis certification, musculoskeletal conditions, orthopaedics, medical cannabis

## Abstract

Background: Amid the ongoing national crisis of opioid misuse in the United States, medical cannabis (MC) has emerged as a potential alternative for chronic pain conditions. This study was performed to understand which orthopedic conditions patients are seeking MC certification for.

Methods: This prospective study was conducted at the Department of Medical Cannabis, Rothman Orthopaedic Institute, Philadelphia, PA, USA. It included consecutive patients with chronic musculoskeletal noncancer pain who were certified for MC, following the Pennsylvania state certification process. Data collected included demographic data, diagnoses, anatomic site of pain, and Patient-Reported Outcomes Measurement Information System (PROMIS) global health scale. The outcome measures from the PROMIS global health scale were used to generate Global Physical Health (GPH) quality of life (QoL) T scores and Global Mental Health (GMH) QoL T scores.

Results: A total of 78 patients were available for analysis following initial MC certification, with 50 (64%) being female and 28 (36%) male. The average age was 63 years with 60% of patients in the 65+ age group. Ethnically, 73 (92%) identified as White, and 70 (90%) were not of Hispanic or Latino origin. The most common reason for seeking MC certification was low back pain (56%), followed by neck pain (21%) and then extremity complaints. The mean GPH QoL T score was 43.71 with a standard deviation of ± 9.86 (p-value = 0.001), while the mean GMH QoL T score was 46.85 with a standard deviation of 8.28 (p-value = 0.0015).

Conclusion: MC cannabis certification was more often sought by women than men and most common for spinal complaints, specifically lower back followed by cervical spine concerns.. This cohort of patients had lower GPH QoL and GMH QoL T scores compared the US general population, representing a significant reduction in the overall physical and mental health.

## Introduction

The United States faces a national crisis from opioid misuse [1]. The utilization of medical cannabis (MC) has emerged as an alternative option for pain relief, potentially alleviating the need for excessive opioid reliance. As of 2023, there are currently 38 states, three territories, and the District of Columbia where MC is legal [2]. As medical and recreational cannabis become increasingly legalized across the United States and in other nations, our understanding of MC continues to expand [3].

MC has shown to be effective in treating chronic noncancer pain, neuropathic pain, seizure disorders, and multiple sclerosis-related spasticities [4-7]. Several randomized control studies have shown a significant analgesic effect of cannabinoids without severe adverse effects [4]. MC has proven to be effective in alleviating orthopedic pain when compared to a placebo [8]. Subsequently, there is a growing interest within the orthopedic field regarding its potential as a treatment or supplementary approach for various painful musculoskeletal conditions [9].

The purpose of this study is to enhance understanding of orthopedic patients’ perspectives on MC by examining the specific orthopedic conditions for which they are pursuing MC certification. This research aims to provide insights into the demographic profiles of patients seeking MC certification. It also explores patients’ perceptions of their overall health including their physical and mental well-being, as assessed through a global health scale [10].

## Materials and methods

This study was conducted at the Department of Medical Cannabis, Rothman Orthopaedic Institute, Philadelphia, PA, USA. Thomas Jefferson University Institutional Review Board (IRB) Committee approved full IRB approval for this project (approval number: 19D.159). All consecutive patients seeking initial MC certification with a diagnosis of chronic musculoskeletal noncancer pain between October 2022 and September 2023 were prospectively enrolled. Patients were enrolled and certified at a single academic practice. 

All physicians participating in MC certification underwent a mandatory four-hour continuing medical education (CME) course and submitted applications to the Pennsylvania Department of Health to become accredited practitioners. All patients applying for MC certification were screened to ensure they met the requirements of being Pennsylvania residents and having one of the 23 state-approved medical conditions [11]. 

Patients eligible for MC underwent a thorough chart review aimed at identifying any history of severe mental health disorders. Those individuals found to have such a history were subsequently excluded. In addition, an evaluation of their prescription drug monitoring history was performed using Pennsylvania’s Prescription Drug Monitoring Program (PDMP)​. In the case that the patient was using opioids, the pain management team was involved to discuss reduction of opioid use to allow for introduction of MC to their regimen. During the certification visit, the chemical components of MC, routes of delivery methods, and optimal dosing parameters were discussed with each patient. Once certified, patients could obtain an MC identification card through the Pennsylvania Department of Health, allowing them to make purchases at state-approved cannabis dispensaries.

For all patients ultimately certified, patient demographics were obtained through the electronic medical records. At the initial visit, the Patient-Reported Outcomes Measurement Information System (PROMIS) global health scale and diagnosis of low back, neck, shoulder, and foot and ankle pain were collected. Due to the variability in the site of musculoskeletal pain, global pain outcome measures were used instead of site-specific disability measures. The PROMIS global health scale generated Global Physical Health (GPH) QoL T scores and Global Mental Health (GMH) quality of life (QoL) T scores [10].

Statistics for GPH and GMH were calculated using the Shapiro-Wilk test. The relationship of explanatory variables with GPH and GMH was tested using T-tests since the outcome variables were normally distributed. P values <0.05 in the analysis were considered statistically significant [10].

## Results

This study included a total of 78 consecutive patients who were eligible and requested MC certification for a musculoskeletal pain diagnosis between October 2022 and September 2023. Of those patients, 28 (36%) were male and 50 (64%) were female. The average age of patients seeking MC certification was 63 years old (range 20-91 years), with the largest number of patients (60%) in the 65+ age group. Ethnically, 73 (92%) respondents identified as White, and 70 (90%) indicated that they were not of Hispanic or Latino origin (Table 1).

**Table 1:** Patient demographics *In the "Race" section, "Other" = one patient identified as African American and one patient identified as Arab

Gender	N	%
Male	28	36%
Female	50	64%
Age		
18-34	4	5%
35-49	9	11%
50-64	18	23%
65+	47	60%
Race		
White	73	94%
Other	2	3%
Declined tosSpecify	3	3%
Ethnicity		
Hispanic or Latino	0	0%
Not Hispanic or Latino	70	90%
Declined to specify	8	10%

**Figure 1:**
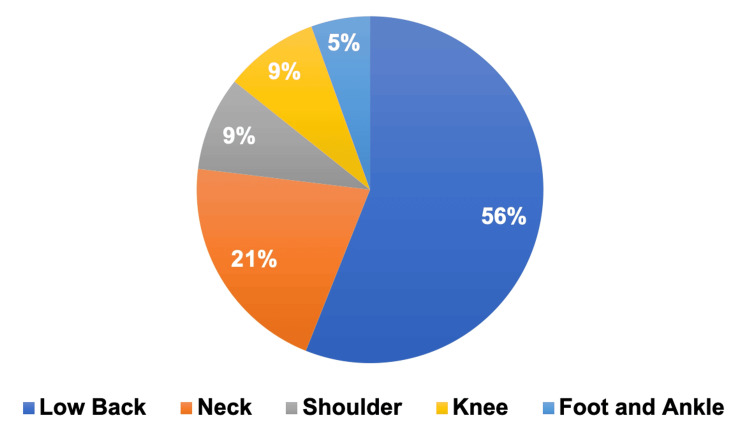
Orthopedic conditions Breakdown of orthopedic conditions for patients seeking medical cannabis certification: low back pain (n = 51), neck pain (n = 19), shoulder pain (n = 8), knee pain (n = 8), and foot and ankle pain (n = 5).

**Figure 2:**
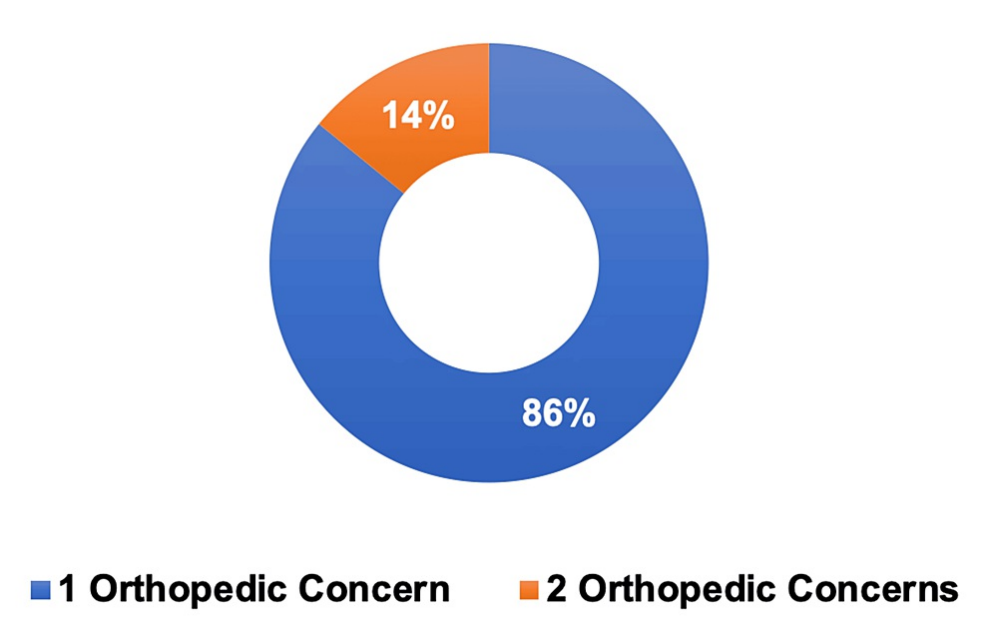
Number of patients with one versus two orthopedic conditions One orthopedic concern (n = 67), two orthopedic concerns (n = 11).

Among the 78 patients with orthopedic concerns, 51 (56%) sought MC certification for low back pain, 19 (21%) for neck pain, eight (9%) for shoulder pain, eight (9%) for knee pain, and five (5%) for foot and ankle pain (Figure 1). Of note, a portion of these patients presented multiple orthopedic concerns, as observed in 11 (14%) patients (Figure 2). 

**Figure 3:**
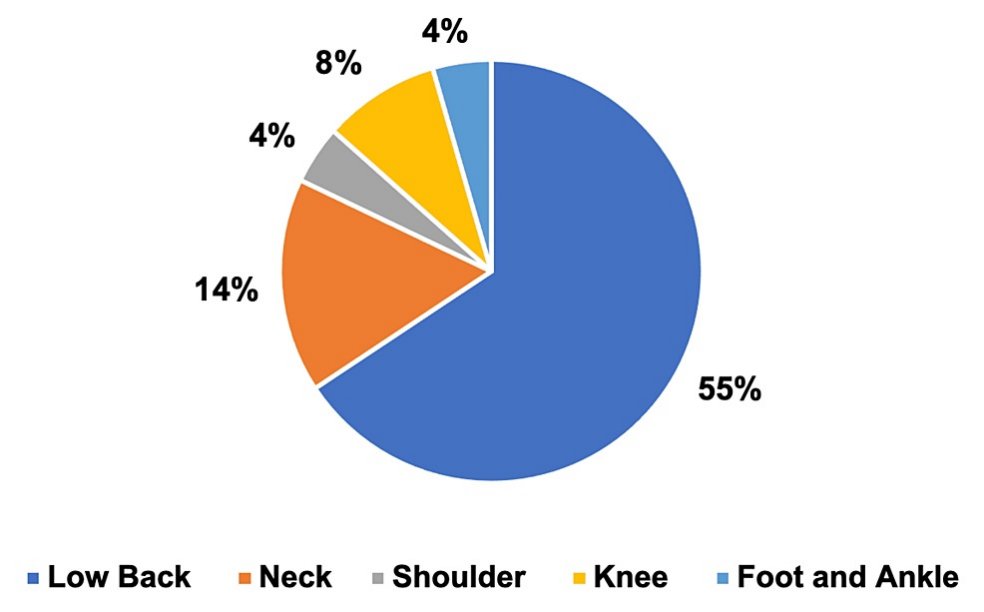
Patients with one orthopedic concern Low back pain = 44, neck pain = 11, shoulder pain = 3, knee pain = 6, foot and ankle pain = 3

Among those with a single orthopedic concern, the largest group consisted of 44 (55%) patients with low back pain, while the smallest group had 3 (4%) patients with foot and ankle pain (Figure 3).

**Figure 4:**
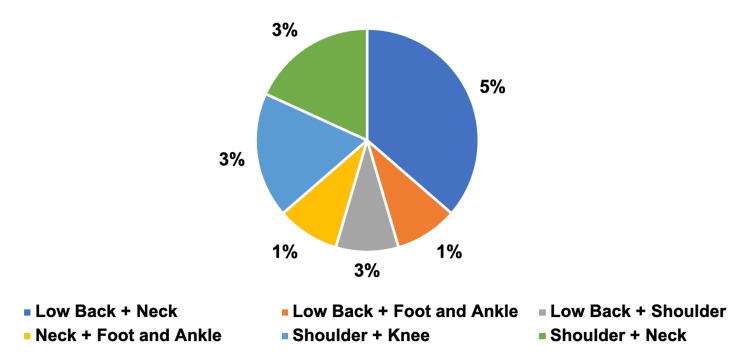
Patient with two orthopedic concerns Low back pain + neck pain = 4, low back pain + foot and ankle pain = 1, low back pain + shoulder pain = 1, neck pain + foot and ankle pain = 1, shoulder pain + knee pain = 2, and shoulder pain + neck pain = 2

For patients with two orthopedic concerns, the most prevalent combination was low back pain and neck pain, with four (5%) patients, while the lowest prevalent combination was observed in low back pain and foot and ankle pain, neck pain and foot and ankle pain, and shoulder pain and neck pain, each with one patient (Figure 4).

**Figure 5:**
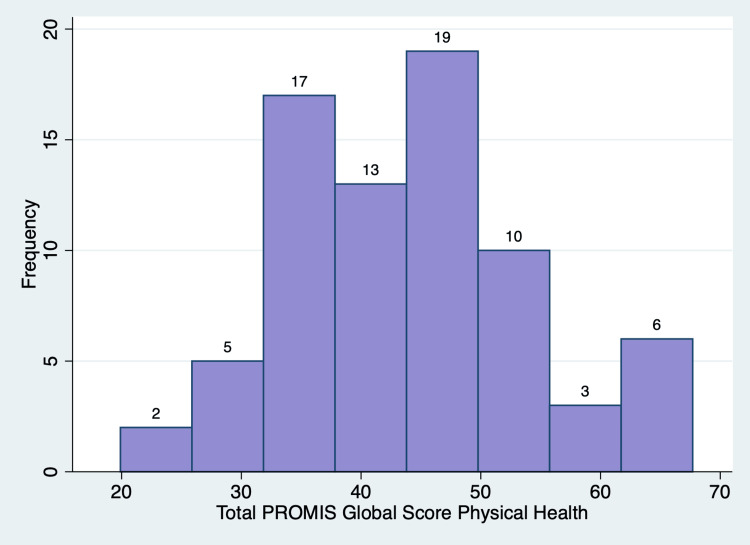
Normal distribution of the Patient-Reported Outcomes Measurement Information System (PROMIS) Global Physical Health T score

**Figure 6:**
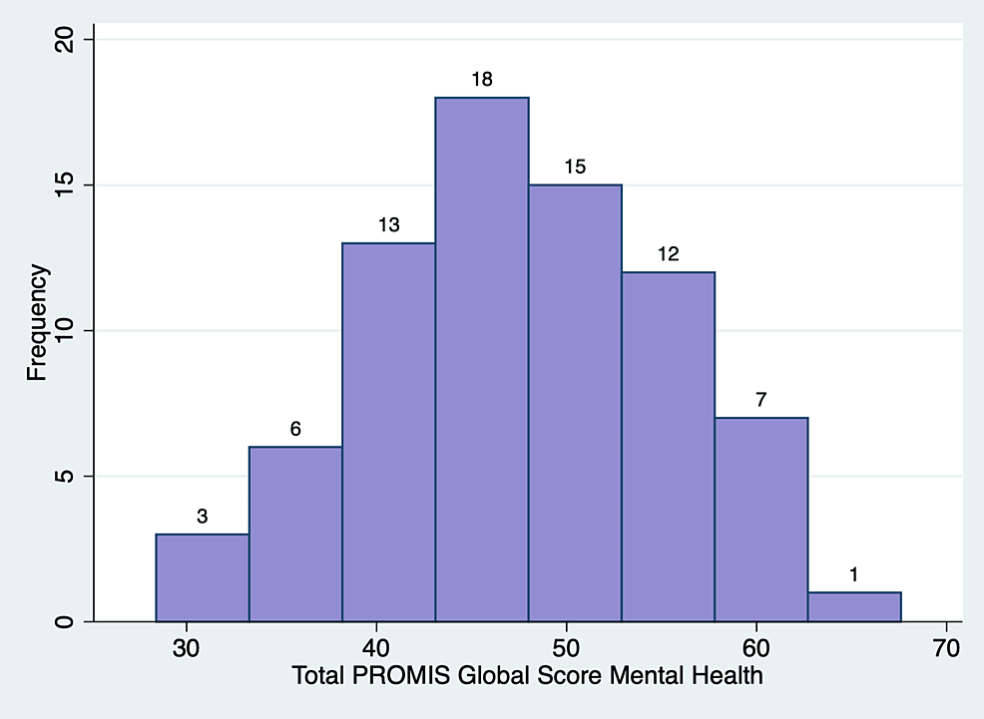
Normal distribution of the Patient-Reported Outcomes Measurement Information System (PROMIS) Global Mental Health T score

PROMIS outcome measures generates T-scores, which are standardized scores with a mean of 50 and a standard deviation of 10 based on a reference population (US general population) [12]. There was a range of responses for each of the items in the PROMIS global health scale (Table 2). For the GPH QoL, the mean T score was 43.71 ± 9.86 (p-value = 0.001), with 56 (72%) patients having lower than the standard T score of 50 (Figure 5). For the GMH QoL, the mean T score was 46.85 ± 8.28 (p-value = 0.0015), with 46 (59%) patients having lower than the standard T score of 50 (Figure 6). Of note, one patient did not fill out a global health survey, while two patients left questions 7, 8, and 10 blank. Thus, these responses were omitted. A posthoc power analysis showed that 78 patients provide 99.68% power to detect one-point change in the PROMIS global health scale (α = 0.05).

**Table 2:** Patient-Reported Outcomes Measurement Information System (PROMIS) global health scale

	Poor (N)		(%)		Fair (N)		(%)		Good (N)		(%)		Very Good (N)		(%)		Excellent (N)		(%)	
1. In general, would you say your health is…	2		3%		23		30%		26		34%		22		29%		4		5%	
2. In general, would you say your quality of life is…	2		3%		25		32%		24		31%		21		27%		5		6%	
3. In general, how would you rate your physical health?	6		9%		29		38%		28		36%		11		14%		3		4%	
4. In general, how would you rate your mental health, including your mood and your ability to think?	2		3%		8		10%		29		38%		25		32%		13		17%	
5. In general, how would you rate your satisfaction with your social activities and relationships?	5		6%		11		14%		22		29%		27		35%		12		16%	
9. In general, please rate how well you carry out your usual social activities and roles.	5		6%		19		25%		25		32%		22		29%		5		6%	
	Not At All				A Little				Moderately				Mostly				Completely			
6. To what extent are you able to carry out your everyday physical activities such as walking, climbing stairs?	3		4%		21		27%		28		36%		20		26%		5		6%	
	1		2		3		4		5		6		7		8		9		10	
7. How would you rate your pain on average?	0	0%	0	0%	3	4%	13	17%	9	12%	13	17%	17	22%	11	14%	7	13%	3	4%
	None				Mild				Moderate				Severe				Very Severe			
8. How would you rate your fatigue on average?	4		5%		18		23%		42		55%		9		12%		2		3%	
	Never				Rarely				Sometimes				Often				Always			
10. How often have you been bothered by emotional problems such as feeling anxious, depressed?	9		12%		24		31%		31		40%		8		10%		2		3%	

## Discussion

This study revealed that patients sought MC certification for a range of musculoskeletal pain conditions, with the most being common lower back pain. This trend is consistent with existing literature as low back pain ranks as the primary cause of years lived with disability in both developed and developing nations, and it holds the sixth position in terms of the overall disease burden [13-15]. Following closely, neck pain was the second most common condition reported. Most patients presented with a single orthopedic concern, but 14% patients presented with dual primary musculoskeletal pain diagnoses, and among this subset, low back pain was still the most prevalent diagnosis.

When examining patient demographics, a larger proportion of women were seeking MC certification compared to men. This observation reflects existing studies, which emphasizes the higher prevalence of chronic back pain among women [16] and the greater likelihood of women to seek medical care for such conditions [13]. Chronic back pain also increases linearly from the third decade of life on, which is observed in the trend of a growing number of patients pursuing MC certification as their age increased [16]. 

The PROMIS global health survey measures an individual’s physical and mental well-being to provide a self-assessment of individuals’ overall health [10]. A higher PROMIS T-score indicates a greater representation of the measured concept [17]. The US general population has a T score mean of 50 and a standard deviation of 10 [12]. An individual with T-scores of 60 for the GPH or GMH scales is one standard deviation healthier than the general population [17]. Patients seeking MC certification had overall lower GPH QoL T scores and GMH QoL T scores with p-values less than 0.05, indicating a significant reduction in both overall physical and mental health compared to the US general population. The patients’ musculoskeletal pain likely contributes to the lower T scores. Importantly, these scores are a baseline measurement as future studies will include this outcome measure to track and examine the effects of MC on patients' self-reported assessments of their overall health.

The existing literature is controversial regarding the impact of MC on opioid use among individuals with chronic pain. While certain studies have demonstrated a reduction in opioid consumption with MC, others have reported no discernible change or even an increase in prescription drug usage [8,18-24]. However, there is a notable lack of research examining the influence of MC on opioid consumption, particularly in the context of chronic musculoskeletal pain [8,24-26]. This study emphasizes the need to further research MC in the realm of chronic musculoskeletal pain. There are limited studies that delineate the patient profiles seeking MC certification. This study clearly shows that patients of both genders and all age groups are considering MC as a component of their pain management strategy for a diverse array of orthopedic conditions.

This study has several limitations. The relatively small number of surveyed patients limits the statistical power of our findings. The patient group is not generalizable evidenced by over 90% of patients identifying as non-Hispanic and Latino and over 90% identifying as White compared to the general US population (58.9% non-Hispanic and Latino and 75.5% White) [27]. Another limitation is the unmeasured variables that cannot be accounted for when studying the relationship between musculoskeletal pain and patients' self-reported assessments of their overall health. These variables could include concurrent medication use, physical activity, and other lifestyle factors. 

Future studies should incorporate follow-up assessments for patients with MC certifications to investigate the different types of cannabis products (whole flowers, oil cartridges, concentrates, tinctures, etc.) they are utilizing, aiming to identify the most effective options. In addition, assessing PROMIS global health scale outcome measures over time could provide valuable insights into the efficacy of MC. Given the limited data available regarding the side effects of various medical cannabis types, it would be insightful to explore the possible adverse effects experienced by patients.

## Conclusions

While MC certification was sought out by patients of both genders and all age groups, it was more frequently sought by women than men and in older ages. The most common complaints pertained to the spine, specifically the lower back followed by cervical spine concerns. This cohort of patients exhibited lower GPH QoL and GMH QoL T scores in comparison to the US general population with p-values lower than 0.05, indicating a significant reduction in both overall physical health and mental health.
